# Empowering Young Physicians’ Contributions in Japan

**DOI:** 10.31662/jmaj.2025-0492

**Published:** 2025-11-21

**Authors:** Soichiro Saeki

**Affiliations:** 1 Department of Emergency Medicine and Critical Care, National Center for Global Health and Medicine, Japan Institute for Health Security, Tokyo, Japan; 2 Division of Public Health, Department of Social Medicine, Graduate School of Medicine, The University of Osaka, Osaka, Japan

**Keywords:** artificial intelligence, interprofessional relations, editorial policies, early-career physicians, editorial fellowship

I would like to sincerely thank Dr. Koga for his thoughtful letter ^(1)^ in response to my recent article on the importance of presenting new ideas and opinions in academic journals ^(2)^. His reflections, drawn from the perspective of a Japanese physician in training abroad, illuminate critical psychological, cultural, and linguistic barriers that many early-career researchers in Japan face when attempting to publish letters or opinion pieces. His observations provide a valuable complement to my initial argument and add important nuance grounded in personal experience.

I fully agree with Dr. Koga that artificial intelligence (AI), particularly large language models, offers promising tools to help overcome linguistic insecurity ^(2)^. Yet, while AI can support clarity and fluency ^(3)^, the generation of genuinely novel ideas remains a distinctly human endeavor. Ideas often arise from lived clinical experience, reflection on social contexts, and―perhaps most importantly―from collaboration with others. Unlike AI, human interactions carry the serendipity and inspiration that frequently spark new insights. Interdisciplinary collaborations, such as those between medicine and engineering, social sciences, or public policy, are particularly fruitful for early-career physicians seeking to broaden their perspectives.

It is therefore essential that Japanese academic medicine foster not only technical tools but also environments that encourage open dialogue, cross-disciplinary exchange, and psychological safety in sharing one’s opinions. Beyond providing guidance and checklists, academic societies and journals must take proactive steps to lower barriers and amplify young voices ^(4)^. One innovative approach is exemplified by the Editorial Fellowship program at *Journal of the American Medical Association* ^(5)^, which allows early-career researchers to participate directly in editorial processes, receive mentorship, and contribute to scholarly discourse. Introducing similar opportunities in Japanese medical journals could empower young physicians by offering both experiential learning and validation of their contributions.

In conclusion, I echo Dr. Koga’s call to address psychological, cultural, and linguistic barriers ^(1)^ while also emphasizing the irreplaceable role of human creativity and collaboration in generating new ideas. By combining supportive environments, appropriate use of AI tools, and institutional initiatives such as editorial fellowships, Japanese academic medicine can motivate young physicians to share their perspectives with confidence and contribute meaningfully to international scholarly discussions.

## Article Information

### Acknowledgments

The author thanks their colleagues for helpful discussions on this topic, especially the Department of Emergency Medicine and Critical Care, International Healthcare Center (ICC) of the National Center for Global Health and Medicine. The author acknowledges the use of Grammarly (Grammarly Inc., San Fransico, USA) for primary language editing. The views expressed in this manuscript are those of the author and do not necessarily represent the author’s institutions. The authors’ institutions played no role in the conceptualization of this manuscript.

### Author Contributions

The author is solely responsible for the contents of this manuscript.

### Conflicts of Interest

None

### IRB Approval Code and Name of the Institution

Not applicable.
